# Quantifying Liver Stagnation Spleen Deficiency Pattern for Diarrhea Predominate Irritable Bowel Syndromes Using Multidimensional Analysis Methods

**DOI:** 10.1155/2018/6467135

**Published:** 2018-02-12

**Authors:** Zhongyu Huang, Zhengkun Hou, Xianhua Liu, Fengbin Liu, Yuefeng Wu

**Affiliations:** ^1^Guangzhou University of Chinese Medicine, Guangzhou, Guangdong, China; ^2^The First Affiliated Hospital of Guangzhou University of Chinese Medicine, Guangzhou, Guangdong, China; ^3^Shenzhen Baoan Traditional Chinese Medicine Hospital Group, Shenzhen, Guangdong, China

## Abstract

**Objective:**

This study aims to offer a new approach for quantifying severity of traditional Chinese medicine pattern with multidimensional analysis methods.

**Methods:**

A scale and theoretical models were constructed based on the definition of liver stagnation spleen deficiency pattern. Clinical data of 344 IBS-D patients from a cross-sectional study was used for feature validation of the model. Confirmatory factor analysis was used for evaluating the models. Also, multidimensional item response model was used for assessing multidimensional psychometric properties of the scale.

**Results:**

Detecting two latent traits, the Cronbach's alpha of the 9-item scale was 0.745. Multidimensional model was evaluated with significant goodness of fit indices while the unidimensional model was rejected. The multidimensional item response model showed all the items had adequate discrimination. Parameters presented adequate explanation regarding mental syndromes having high factor loading on the liver stagnation factor and abdominal discomfort syndromes highly related to the spleen deficiency factor. Test information function showed that scale demonstrated the highest discrimination power among patients with moderate to high level of severity.

**Conclusions:**

The application of the multidimensional analysis methods on the basis of theoretical model construction provides a useful and rational approach for quantifying the severity of traditional Chinese medicine patterns.

## 1. Introduction

Diarrhea predominate irritable bowel syndromes (IBS-D) is one of the most commonly seen diseases in traditional Chinese medicine (TCM) clinical practice. With complex clinical appearance and integrated affect, IBS-D has both psychological and physical effects on patients, which causes deterioration in their quality of life [1]. TCM approach has been reported as an effective alternative therapy in relieving related discomforts caused by IBS-D [2–4]. TCM pattern is defined as a summary of disharmony condition of body elements based on prestigious TCM theory such as Yin-Yang and Wu-Xing theory. Accurate pattern diagnosis of patients assists in a better treatment of disease [3, 5]. However, traditional approaches of pattern diagnosis subjectively rely on individual experience of the practitioner and there is no yet adequate evidence found for objective detection of TCM pattern.

The symptoms of IBS-D patients involve several systems, which is complex to make complete assessment for comprehensive evaluation of each patient. In recent years, methods of interdisciplinary research such as computer science and psychology have been imported and applied in disease detection and longitude management [6, 7]. Instruments with quantitative characteristics such as IBS-SSS [8] and IBS-QOL [9] have been developed for diseases evaluation or quality of life measurement for IBS. However, those scales are not suitable for TCM pattern detection due to differences between their theories. As far as TCM pattern is concerned, theoretical interactions between symptoms and pattern factor are complex with multidimensionality. Thus, instruments developed in classical test theory with linear unidimensional assumption do not meet the requirement for quantitative analysis of pattern. Overall, as to IBS-D, there is a lack of instruments specially developed for quantitative severity detection of TCM pattern.

Against high-dimensionality assumption of the real-world, factor analysis methods such as exploratory factor analysis (EFA) and principal component analysis are commonly used to construct and simplify the theoretical model by finding out latent factors from observed variables. However, there are limitations about these methods in linear assumption because most of the systems including disease are inherently nonlinear.

Latent variable model was reported to be useful for quantifying TCM patterns by estimating nonlinear relationship between individual ability and responses about severity of symptoms and signs [10, 11]. In this study, we attempted to present a latent trait analysis process for quantitative analysis of multidimensional TCM pattern by forming and validating a scale for the pattern severity detection of IBS-D with the hope of assisting patterns detection and efficacy estimation in TCM clinical practice.

## 2. Methods

### 2.1. Data Source

Cross-sectional study design was used. Patients aged 18–80 from the first affiliated hospital of Guangzhou university of Chinese medicine with a previous clinical diagnosis of IBS-D were assessed by the scale between January 2015 and March 2017. All patients were diagnosed by senior clinical experts referring to the Roman III classification criteria for IBS-D.

According to the Guidance of Chinese medicine for prevention and treatment for digestive disease [3], liver stagnation spleen deficiency pattern could be diagnosed with the presence of at least two primary symptoms and two secondary symptoms as follows: primary symptoms: diarrhea with abdominal pain that induced or aggravated by mental disorders, sensation of contracture of the lower abdomen, and abdominal pain and fullness sensation in chest-hypochondriac region, stringy pulse; secondary symptoms: intestinal flatus from anus, mucous stool, depression, or impetuosity.

Exclusion criteria were set as follows: any patient who does not meet the inclusion criteria; any patient who does not sign the informed consent form; any case whose questionnaire is incompletely filled (the absence and omitting of self-administered items except general information should not beyond 5% or no interview); and any patient who catches metabolic syndrome.

### 2.2. Tool and Measurement

**Figure 1:**
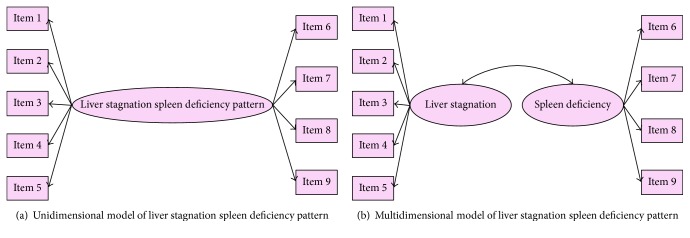
Construction of theoretical models for liver stagnation spleen deficiency pattern. Items 1–9 were listed in the scale representing symptoms or signs related to the pattern.

Related symptoms or signs of the pattern were picked to build the theoretical model. None of the symptoms or signs was shared between syndromes. Besides, signs of tongue and pile were not included in the model because of lacking objective criteria for quantitative collection. According to modification advice of TCM experts, the theoretical pattern model of two factors was finally set up as shown in Figure 1 together with the unidimensional model.

A 9-items scale was developed under the supervision of senior TCM experts to evaluate the severity of liver stagnation spleen deficiency pattern of IBS-D. As shown in Table 1, item options are designed with four categories indicating four increasing levels of severity of symptoms by “none,” “mild level,” “middle level,” and “serious level.” Two clinical experts with senior title were invited to evaluate patients through interviews, so as to reduce the errors of the severity detection in the assessment and strengthen the accuracy of pattern diagnosis. Data collected in the assessment were then analyzed using a mixture of methods of latent variable analysis and classical statistical analysis.

### 2.3. Statistical Methods

Descriptive statistics was performed to analyze demographic characteristics of the enrolled patients in SPSS 22. Psychometric properties including content validity and reliability as well as construct validity were analyzed by integration of classical test theory and modern test theory.

Content validity was evaluated to test whether the involved items measure the concept adequately and sufficiently. Consistence of the items in the instrument was measured by Cronbach's alpha coefficient in SPSS 22. Confirmatory factor analysis (CFA) was used for measuring structural validity of the instrument. Parameters of the analysis were estimated with maximum likelihood method in AMOS 21. The comparative fit indices (CFI) and the root mean square error of approximation (RMSEA), which were widely used and known to be relatively unaffected by sample size [12], were picked as judge of practical fit. It indicates a reasonable approximation of reality with RMSEA being equal to or less than 0.05 and the CFI approaching 1 [13].

Multidimensional graded response model (MGRM) was used for fitting the multidimensional theoretical model with the polytomous clinical data. With the assistance of packages including mirt [14] and mirtCAT [15] in R 3.4.1. Quasi-Monte Carlo EM estimation [16] was used as algorithm for MIRT parameters calculation of each item. As defined in formula (1), giving the ability vector *θ*, *p*_*jt*_ is defined as probability for the examinee with ability *θ* to make correct response by selecting the category *t* (and above) of item *j*. Parameter *a*_*j*_ represents the discrimination vector of item *j*, and *d*_*j*_ is the item-category parameter vector. Thus, the probability of making exact selection with the *t-*category (and above) of item *j* could be calculated by *p*_*jt*_ minus *p*_*jt*−1_ as shown in formula (2). In this study, the severity of the pattern consisting of two factors could be assessed as the *θ* with response to the item since discrimination and item-category parameters are estimated:

(1) pjt∗=∫−∞ajTθ+djt12Πe−x2/2dx,t=1,2,…,mj,

(2) pjt=pjt∗−pj,t+1∗∫ajTθ+dj,t+1ajTθ+djt12Πe−x2/2dx,t=1,2,…,mj.

MDISC (multidimensional discrimination index) was calculated to give an overall measurement of the quality of each item [17]. An item is considered as high quality with its MDISC higher than 1, while an item is considered as medium quality with its MDISC between 0.5 and 1. Item information function is a direct reflection of estimation accuracy of the assess scores for examinee's ability. In the current study, item information of each item was calculated to evaluate precision of the item in pattern severity assessment. Test information surface and item trace surfaces together with item character curves (ICCs) were plotted in R as intuitive evaluation references for precision of the assessment and rationality of the item setting.

## 3. Results

A total of 368 patients were diagnosed with IBS-D by both Rome III criteria and TCM criteria. 22 patients were excluded, among which 5 did not completely fill the questionnaire; the others rejected to participate in the project.  Table 2 showed the demographics and characteristics of the 344 patients of IBS-D. The median age was 38 years old, and the youth took the greatest part (60.46%) among the sample while the old taking the least (7.56%).

Cronbach's alpha coefficient of the whole items of the scale was 0.745. Unidimensional model and multidimensional model were evaluated with CFA and the indices of both model fit are presented in Table 3. It reported that the unidimensional model was rejected by the Chi-square test with *p* < 0.05. Moreover multidimensional model displayed a better goodness of fit with RMSEA = 0.03 and CFI = 0.98.

Factor loading of each item on its related factor together with MIRT parameter of all items is shown in Table 4. Most of the items had at least a moderate loading on their related factors. However, item 4 and item 8 representing symptoms “sensation of contracture of the lower abdomen” and “anorexia” had factor loading lower than 0.4.

Full-information item factor analysis of the multidimensional model converged within 0.0001 tolerance after 142 EM iteration. All items have at least an acceptable discrimination power (MDISC > 0.5) and some have a very high discrimination power with MDISC over 2.0. Item-category parameters showed descending trend as categories increased showing consistency with theoretical setting of MGRM.

**Figure 2:**
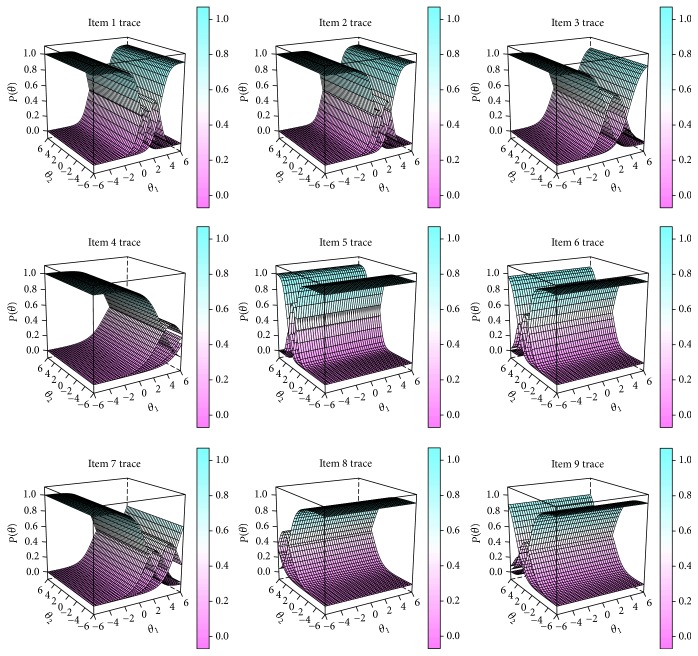
Item trace surfaces of the scale.

**Figure 3:**
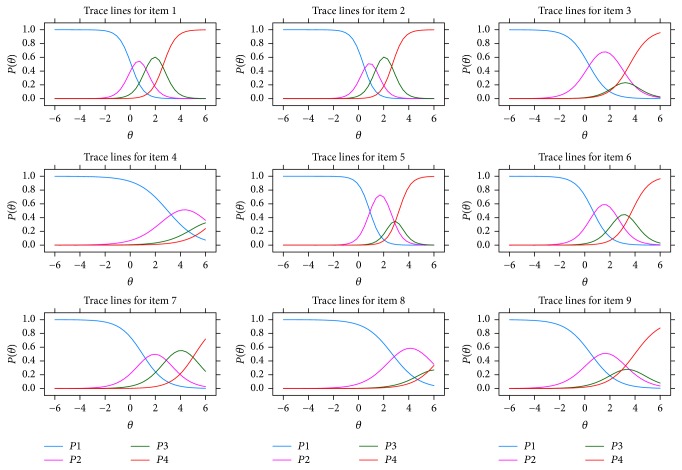
Trace lines for 9 items of the scale.

Item trace surfaces were plotted to describe characters of item setting for the two-factor assessment in Figure 2. Showing more details, trace lines for each item were plotted as item character curves (ICCs) on related factor in Figure 3. All items except item 4 and item 8 showed typical ICCs with the curve of the first category and last category in monotonicity and others in normality. For the two items, the tops of the arc for the four character curves were not significantly separated on the axis *θ*. Also, both of the curves even showed overlapping trends as the *θ* parameter increased. ICCs of the two items indicated that category gradients of these items should be modified to match the grading of response from patients with different level of severity.

**Figure 4:**
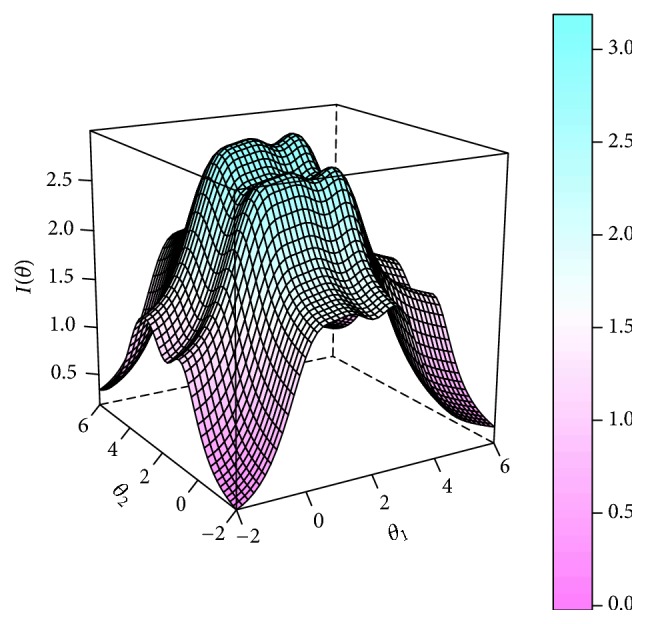
Test information function surface of the scale.

Test information function surface indicates the precision of assessment. As shown in Figure 4, with the hump of surfaces ranging between −2 and 6, the scale could well discriminate the severity of the pattern among patients with moderate to serious condition. Also, it is most discriminative for those with severity of the pattern at the range of 0 to 3 as the peak of the surface but becoming useless for extreme cases with minimum or maximum severity.

## 4. Discussion

In this study, a scale with 9 items was designed for quantifying severity of liver stagnation spleen deficiency pattern for IBS-D patients. Using data collected from 344 patients, reliability of the scale was validated with Cronbach's alpha coefficients over 0.7. Compared to the unidimensional model, multidimensional model was evaluated with better indices of model fit. Two factors of the model representing liver stagnation and spleen deficiency were evaluated with significant association with the responses of items by MIRT which offered convincible interpretation for the theoretical model. Overall, validation and reliability of the scale were evaluated to be adequate. The scale provided most information of the patients with the severity of pattern in moderate level. Most items were evaluated to be rational with convincible MIRT parameters and information curves.

Reliability of the scale evaluated by Cronbach's alpha coefficient indicates internal consistency for scale-level measurement while that for item-level measurement is not specified. Generally, test-retest reliability and alternate-form reliability are used as complementary method for detailed evaluation. In this study, only the Cronbach's alpha coefficient was calculated because there were restrictions that made it difficult to carry out the other two methods. TCM pattern is defined as diagnostic summary of the pathological changes of a disease state equipping pattern with dynamic feature. Steady condition of the pattern is hard to assess to evaluate the test-retest reliability. Moreover, it is impossible to carry out alternate-form reliability evaluation in this study considering compliance of the participants.

In CFA, the multidimensional model represented better goodness of fit to the response data than that of the unidimensional model. Also, unidimensional assumption is not suitable for simultaneous measurement of multiple dimensions. Thus, the MIRT model of MGRM was used for the scale calibration. Representing distinct factors of “liver stagnation spleen deficiency” pattern, the two latent traits were correlated to each other according to TCM theory. Liver governs conveyance and dispersion while spleen deficiency factor representing movement and spleen governs the pivot of qi. Liver stagnation leads to a disorder of the pivot of qi, thus affecting the function of movement and transformation governed by spleen. Since subjective evidence of the theory had not been found, one-one correlation was designed between item and latent trait with idealization so as to simplify the assessment of pattern. Many items had very high discrimination parameters. The test information function surfaces showed that the scale was most discriminative among patients with moderate severity of the pattern. It is consistent with TCM clinical practice that detectable typical syndromes are more valuable for pattern differentiation. Moreover, it is easier to make a diagnosis as the severity of disease increased.

Correlation parameters of item-to-item and item-to-factor were significant, and quantitative model could be well explained in theory. Evidence was found in previous study that IBS is commonly triggered by underlying psychological conditions such as trauma, depression, and anxiety. Also, TCM theory describes the mechanism in a different way. According to TCM theory, liver plays an important role in the occurrence and regulation of psychological stress. Depression and irascibility lead to stagnation of liver, which disturbs the transportation function governed by spleen and thus may cause disorder in digestion. With mental related symptoms taking the greatest weights on the liver stagnation spleen deficiency pattern, the estimated parameters of the items could also be well explained.

This study has several limitations. Firstly, although the psychometrics properties of the scale estimated in MIRT were adequate, MIRT model used for measuring multidimensional constructs required larger sample size (*n*⩾500) than we had in this study. Therefore, a study with larger sample needs to be carried out for reevaluating the scale. Secondly, even though construct validity of the scale was approved in CFA, it is not proved whether the scale could make comprehensive assessment of the pattern without considering other symptoms or signs related to IBS-D. Thirdly, both of the setting of some items and standardized criteria rules for the interview should be modified for improving the assessment that could be affected by the different understandings between examinees toward the definition of item. Finally, since the scope of study was limited, multicenter study is to be carried out for more persuasive conclusion before practical application in clinical environment.

Regardless, this study provides a new example for quantifying TCM pattern with a combined application of classical test theory and modern test theory. Regarding the multidimensionality of TCM pattern, CFA and MIRT were integrated and used as a new approach of severity assessment. Though previous study had made use of modern test theory for pattern analysis, importance of construct validity was always ignored. Rather than making complementary explanation of the result, this study formed and resolved the scale under guidance of TCM theory innovatively. Though the technique does not solve the pseudoscience controversy toward TCM, the method would be still helpful for complex structural model analysis such as pattern.

## 5. Conclusion

In this study, a scale with 9 items designed for severity assessment of “liver stagnation spleen deficiency” pattern of IBS-D was validated based on a multidimensional model. With good discrimination power, the scale could be further used as assisting tool for pattern detection in TCM practice after reevaluation and optimization. Multidimensional analysis methods provide a practical and rational approach for quantifying TCM pattern. Further research should be carried out to reevaluate the scale so as to provide practical instrument to assist with pattern detection and therapeutic efficacy measurement for TCM clinical practice.

## Figures and Tables

**Table 1:** Scale for assessment of liver stagnation spleen deficiency pattern of diarrhea predominant irritable bowel syndrome.

Content	Definitions of item categories			
	0	1	2	3
(1) Irritability and susceptibility to rage	Negative	Slightly	Moderately	Severely
(2) Depression	Negative	Slightly	Moderately	Severely
(3) Discomfort aggravated by mental disorders	Negative	Slightly	Moderately	Severely
(4) Sensation of contracture of the lower abdomen	Negative	Slightly	Moderately	Severely
(5) Sigh	Negative	Occasionally	Sometimes	Always
(6) Gastrointestinal rumbling	Negative	Occasionally	Sometimes	Always
(7) Intestinal flatus from anus	Negative	Slightly	Moderately	Severely
(8) Anorexia	Negative	Slightly	Moderately	Severely
(9) Discomforts alleviated after defecation	Negative	Slightly	Moderately	Always

**Table 2:** Demographics and characteristics of the 344 enrolled IBS-D patients.

Variables	Total (*n* = 344)	Percentage
*Gender*		
Male	180	52.33%
Female	164	47.67%
*Age *		
Youth (18–40)	208	60.46%
Middle-age (41–65)	110	31.98%
Old-aged (>65)	26	7.56%

IBS-D: diarrhea predominant irritable bowel syndrome.

**Table 3:** Indices for liver stagnation spleen deficiency pattern models (*N* = 344).

Indices	Unidimensional model	Multidimensional model
CFI	0.93	0.98
RMSEA	0.06	0.03
AIC	112.48	89.79
*χ* ^2^	58.48	33.79
Df	27	26
*p*	*∗∗*	0.14

CFI: comparative fit index. RMSEA: root-mean-square error of approximation. ^*∗∗*^*p* < 0.01.

**Table 4:** Estimation of factor loadings and MIRT parameters of items.

Items	Factor loadings		MIRT parameters					
	*f*_1_ (SE)	*f*_2_ (SE)	*a*_1_	*a*_2_	*d*_1_	*d*_2_	*d*_3_	MDISC
1	0.69 (0.04)^*∗*^	0	2.03^*∗*^	0	−0.15^*∗*^	−2.60^*∗*^	−5.39^*∗*^	2.03^*∗*^
2	0.71 (0.05)^*∗*^	0	2.19^*∗*^	0	−0.83^*∗*^	−3.10^*∗*^	−5.91^*∗*^	2.19^*∗*^
3	0.55 (0.06)^*∗*^	0	1.29^*∗*^	0	−0.39^*∗*^	−3.71^*∗*^	−4.65^*∗*^	1.29^*∗*^
4	0.30 (0.08)^*∗*^	0	0.84^*∗*^	0	−2.51^*∗*^	−4.78^*∗*^	−6.19^*∗*^	0.84^*∗*^
5	0.43 (0.07)^*∗*^	0	1.12^*∗*^	0	−1.10^*∗*^	−3.29^*∗*^	−5.78^*∗*^	1.12^*∗*^
6	0	0.60 (0.07)^*∗*^	0	2.21^*∗*^	−1.95^*∗*^	−5.66^*∗*^	−7.11^*∗*^	2.21^*∗*^
7	0	0.62 (0.05)^*∗*^	0	1.48^*∗*^	−0.95^*∗*^	−3.67^*∗*^	−5.59^*∗*^	1.48^*∗*^
8	0	0.28 (0.07)^*∗*^	0	0.94^*∗*^	−2.51^*∗*^	−5.19^*∗*^	−6.30^*∗*^	0.94^*∗*^
9	0	0.54 (0.06)^*∗*^		0.98^*∗*^	−0.49^*∗*^	−2.74^*∗*^	−3.88^*∗*^	0.98^*∗*^

SE: standard error. *f*_1_: liver stagnation factor. *f*_2_: spleen deficiency factor. *a*_*i*_: discrimination parameter of dimension *i*. *d*_*j*_: easiness parameter of category *j* of an item. MDISC: multidimensional discrimination parameter of item. ^*∗*^*p* < 0.05.
